# An evidence synthesis of the international knowledge base for new care models to inform and mobilise knowledge for multispecialty community providers (MCPs)

**DOI:** 10.1186/s13643-016-0346-x

**Published:** 2016-10-01

**Authors:** Alison Turner, Abeda Mulla, Andrew Booth, Shiona Aldridge, Sharon Stevens, Fraser Battye, Peter Spilsbury

**Affiliations:** 1 Strategy Unit, Midlands and Lancashire Commissioning Support Unit, West Bromwich, UK; 2 School of Health and Related Research, University of Sheffield, Sheffield, UK

**Keywords:** Primary health care, Innovation, organizational, Community health services, Integrated health care

## Abstract

**Background:**

NHS England’s Five Year Forward View (NHS England, Five Year Forward View, 2014) formally introduced a strategy for new models of care driven by simultaneous pressures to contain costs, improve care and deliver services closer to home through integrated models. This synthesis focuses on a multispecialty community provider (MCP) model. This new model of care seeks to overcome the limitations in current models of care, often based around single condition-focused pathways, in contrast to patient-focused delivery (Royal College of General Practitioners, The 2022 GP: compendium of evidence, 2012) which offers greater continuity of care in recognition of complex needs and multimorbidity.

**Methods:**

The synthesis, an innovative combination of best fit framework synthesis and realist synthesis, will develop a “blueprint” which articulates how and why MCP models work, to inform design of future iterations of the MCP model. A systematic search will be conducted to identify research and practice-derived evidence to achieve a balance that captures the historical legacy of MCP models but focuses on contemporary evidence. Sources will include bibliographic databases including MEDLINE, PreMEDLINE, CINAHL, Embase, HMIC and Cochrane Library; and grey literature sources. The Best Fit synthesis methodology will be combined with a synthesis following realist principles which are particularly suited to exploring what works, when, for whom and in what circumstances.

**Discussion:**

The aim of this synthesis is to provide decision makers in health and social care with a practical evidence base relating to the multispecialty community provider (MCP) model of care.

**Systematic review registration:**

PROSPERO CRD42016039552.

**Electronic supplementary material:**

The online version of this article (doi:10.1186/s13643-016-0346-x) contains supplementary material, which is available to authorized users.

## Background

NHS England’s Five Year Forward View [1] formally introduced a strategy for new models of care driven by simultaneous pressures to contain costs, improve care and deliver services closer to home through integrated models. The combined aims of these new care models are to reduce the high level of avoidable admissions [2] and offer improved quality, outcomes and patient satisfaction [1, 3–6].

Following a NHS England call to register interest in delivering new care models for three types of “Vanguards”: multispecialty community providers (MCPs), care homes and integrated primary and acute care systems in January 2015 [7], the first wave of Vanguard sites were selected in March 2015. Further waves, focusing on urgent and emergency care (http://www.england.nhs.uk/ourwork/futurenhs/5yfv-ch3/new-care-models/uec/) and acute care collaborations, followed later in the year (http://www.england.nhs.uk/ourwork/futurenhs/5yfv-ch3/new-care-models/acute-care-collaboration/).

Our evidence synthesis focuses on multispecialty community providers (MCPs); the expectation is that MCPs will eventually become integrated providers of out-of-hospital care. This new model of care seeks to overcome the limitations in current models of care, often based around single condition-focused pathways, in contrast to patient-focused delivery [8] which offers greater continuity of care in recognition of complex needs and multimorbidity. The case for integrated care is reinforced by the need to develop whole-system working [9] which includes greater patient and community involvement [10]. The MCP models of care therefore signal a growing shift towards greater community involvement and collaboration with the voluntary sector.

## Methods and design

### Objectives

The aim of this synthesis is to provide decision makers in health and social care with a practical evidence base relating to the multispecialty community provider (MCP) model of care. The synthesis, based on realist principles, will develop a “blueprint” which articulates how and why MCP models work, to inform design of future iterations of the MCP model. We believe this synthesis will support decision makers in a range of settings, by:

- Supporting the required local evaluation of the MCP sites
- Enabling shared learning for the design and delivery of the MCPs in a timely way
- Informing policymakers of the “active ingredients” for successful new models of care

Specifically, the objectives of the synthesis are to:

- Articulate the underlying programme theories (what it is about each programme which will result in the desirable change/s) of the MCP model of care, by mapping the logic models of the 14 MCP demonstrator sites, alongside other key documentation, prioritising key theories for investigation
- Identify sources of theoretical, empirical and practice evidence to test the programme theories identified
- Appraise, extract and analyse evidence, reconciling confirmatory and contradictory evidence
- Develop the synthesis, producing a “blueprint” to explain how the mechanisms used in different contexts contribute to outcomes and process variables
- Consult with key stakeholders from selected MCP demonstrator sites to validate findings and to test applicability to different contexts
- Finalise the synthesis, incorporating stakeholder feedback
- Disseminate the findings, preparing a series of practical tools to support knowledge mobilisation

A full list of programme theories, interventions and mechanisms will be extracted from the logic models developed by the Vanguards [11]. In accordance with our identified methodology, we will identify an a priori “best fit” framework which is both relevant to the context of integrated health and care services and meaningful to the intended audience for our final synthesis. We will use the a priori framework to code underlying programme theories of the current MCP Vanguards and mechanisms identified. These will be prioritised with the support of the Project Advisory Group. The logic models developed by the Vanguards typically follow the model recommended in the Magenta Book [12] which lends itself to the CIMO (context, intervention, mechanisms, outcomes) [13] framework with which we intend to plan the search strategy.

### Review question(s)

- What are the foremost theories of change inherent within the MCP model of care?
- What seem to be the “active ingredients” which should inform design of MCP models of care?
- What are the social and cultural conditions which influence (enabling and blocking) change within MCP models of care and how do these mechanisms operate in different contexts?
- What are the key knowledge gaps and uncertainties in relation to the design, implementation and evaluation of MCP models of care?

### Search process

A systematic search will be conducted to identify research and practice-derived evidence between January 2000 and December 2016. This will achieve a balance that captures the historical legacy of MCP models but focuses on contemporary evidence. Sources will include bibliographic databases including MEDLINE, PreMEDLINE, CINAHL, Embase, HMIC and Cochrane Library; and grey literature sources such as the King’s Fund and Nuffield Trust.

Candidate search terms will be identified by analysing documentation, including logic models, from the current MCP demonstrator sites. These will be reviewed by the project team, with particular support from the Vanguards Relationship Lead; we will also seek advice from the Advisory Group setup to support the synthesis. The Advisory Group represents key stakeholder groups, with membership including senior leaders from three MCP Vanguards, a community NHS Trust, a Clinical Commissioning Group and NHS England; members of the public; and the University of Sheffield (the objectives of the Group are included as an Additional file 1).

Given the difficulties in searching for evidence in social science fields [14], particularly with regard to concepts related to integrated care (i.e. inconsistent definitions, changing terminology), we will check the reference lists of included evidence, to identify additional evidence. As evidence is likely to be distributed across a wide variety of professional and managerial evidence sources, we do not plan to use formal hand searching of a select list of resources. Instead, we will systematically follow up citation networks using both the Google Scholar “cited by” function and formal Web of Science citation searching.

The scope of the search will include international literature within a developed country context to ensure inclusion of relevant literature of appropriate mechanisms—defined as [15]: “the interaction between what the programme provides and the reasoning of its intended target population”. For example, a model based on mutuality, perceiving service users/patients as partners involved in decision making might involve a range of mechanisms relating to relationships with local communities. However, we acknowledge that findings from other health systems do not always transfer well to NHS settings [16] and may yield indicative rather than definitive findings.

We do not propose to include non-English language studies for the following reasons:

- Our methodology incorporates realist principles which emphasise the importance of context, privileging relevance over rigour. Although non-English papers will address some activities and mechanisms relevant to MCPs, it is the combination of those activities and mechanisms within the NHS setting which is particularly important. Including non-English papers would compromise the fidelity of the synthesis to the target setting/context.
- Logistically, including translation of papers would add both time and costs, for arguably little additional value.

Based on early plans of existing MCPs, we have identified a core set of potential search terms which capture the common features and activities of MCP models of care (these are included as an Additional file 2).

We have described our methods as per Preferred Reporting Items for Systematic Review and Meta-Analysis for protocol (PRISMA-P) recommendations [17], and this checklist is included as an Additional file 3.

### Types of study to be included

We aim to identify relevant evidence on the most significant (prioritised with our Advisory Group) contexts, mechanisms and outcomes relevant to the MCP demonstrator sites. As it is likely that there will be, as yet, little evidence explicitly on UK MCP models of care, we propose to explore the literature which could be described as the “intellectual heritage” of the MCP model (e.g. [18, 19]).

In terms of defining evidence, decision makers in large-scale change programmes need to draw on multiple types of evidence. We will adopt Williams and Glasby’s [20] definition which describes “evidence” from an evidence-based management rather than evidence-based medicine perspective, comprising empirical evidence from research; practice-based and experiential evidence from service delivery; and theoretical evidence. Our search strategy therefore will include research studies (trials and reviews), service evaluations and case studies, in addition to thought-leading papers.

We will focus on a core of highly relevant literature, using this to identify secondary terms and other relevant papers, in an iterative cycle. This approach, which is well accepted within a realist synthesis context [21], is methodologically stronger than an optimally sensitive search strategy which runs the risk of identifying such a large volume of marginally relevant and irrelevant papers, that time for analysis will be compromised by time spent sifting candidate references.

### Types of population

The population targeted by the evidence synthesis will include patients, carers and communities receiving care via interventions which feature in the MCP model of care. We will also focus on staff across health and social care involved in the design, delivery and evaluation of interventions delivered within the MCP model of care.

### Types of intervention

The synthesis will focus on the 14 MCP Vanguards within the NHS England’s Vanguards programme. These MCP Vanguards aim to develop extended primary care services, “offering some care in fundamentally different ways, making fuller use of digital technologies, new skills and roles, and offering greater convenience for patients”, essentially becoming “the focus of a far wider range of care needed by their registered patients” [1]. Primary care is a priority development area for local health economies, driven by the shift towards co-commissioning, integrated care and patient empowerment.

The MCP models of care aim to provide wrap-around and coordinated services for patients, which, whilst following some standard principles, will adapt to fit with the local context. The evidence base on system integration is variable, characterised by a lack of consensus on what constitutes integrated care [22–24]. It is generally accepted that no single model or approach to integrated care can be applied universally [25, 26] and this is evident in the models developed in the 14 MCP demonstrator sites. A rapid desk-based analysis of the aims and objectives of the MCP demonstrators suggests that these models of care incorporate a number of different interventions at different levels (micro, meso and macro) within the system, including extensivist primary care services, multidisciplinary case management and social prescribing.

### Types of outcome

This study will review evidence relating to the stated outcomes of MCP models, as articulated in the MCP logic models. These relate to the quadruple aim: patient experience, the health of the population, healthcare costs and staff experience. The synthesis will include qualitative data and quantitative measures.

### Data extraction and analysis

Retrieved papers will be managed using Endnote and will be screened by the Chief Investigator and Vanguards Relationship Lead, for relevance according to the mechanisms identified and prioritised in the a priori framework.

We anticipate including all evidence deemed to have satisfied the relevance criteria; several researchers (e.g. [27]) contend that exclusion of qualitative research on the basis of quality risks missing important insights. Studies will be appraised using standard tools [28, 29].

Data will be extracted using a standard form to capture key characteristics necessary to understand the context of the evidence; relevant concepts identified from the a priori framework and which have emerged from the evidence; and important findings on mechanisms and outcomes. Thematic analysis across the selected evidence base will identify confirming and conflicting theories which will form the basis of a draft conceptual model. NVivo will be used to manage data analysis.

To illustrate how the “best fit” framework analysis will work in practice, we conducted a rapid desk-based analysis of the MCP Vanguard applications which suggests multiple interventions and mechanisms are being developed at micro, meso and macro levels, e.g. extensivist primary care, multidisciplinary community teams, social prescribing (interventions), community assets and social capital (mechanisms). A number of potential themes also feature in guidance [11] from NHS England: design, evaluation, integrated commissioning, patient and community empowerment, technology, workforce, leadership and engagement.

Potential “umbrella” mechanisms and themes will be used as a framework for data extraction against which the literature on new models of care will be extracted and analysed. Within each of these umbrella mechanisms, we will derive if-then causal statements, based on the literature, that seek to unpick what exactly it is about these mechanisms that is likely to result in improved outcomes or process variables. For example we will be able to identify the putative active ingredients for extensivist services, accountability, reduced fragmentation etcetera that likely result in the intended (or indeed unintended) outcomes. We will seek preliminary verification of these if-then statements with a convenience sample of staff working within existing Vanguard sites. This convenience sample will be recruited from the current Vanguards; we will aim to represent a minimum of five different sites.

We will seek to verify the draft conceptual model with key stakeholders from within the MCP Vanguards and NHS England. This will be achieved via the project’s Advisory Group and a dedicated focus group with key stakeholders. This is an important phase of the project, ensuring that the model is fit for purpose and meaningful to decision makers and practitioners.

### Risk of bias (quality) assessment

As the MCP model comprises a broad range of activities and mechanisms, the potential evidence base is vast and diffuse. We anticipate that the main types of evidence sources identified will be of four main types:

1. Before/after studies or interrupted time series from previous or current initiatives (i.e. MCPs or forerunners such as accountable care organisations (US))
2. Descriptive reports/case studies of current MCP initiatives, offering contextual detail and programme theory
3. Commentaries, editorial and opinion pieces on the characteristics and rationale of MCPs and their forerunners
4. Policy documents (e.g. from NHS England)

Only category 1 material is suitable for formal risk of bias assessment. Quality appraisal will be performed on a case-by-case basis using appropriate tools from the Cochrane Effective Practice and Organisation Group (EPOC) to examine bias in this category of studies (*Suggested risk of bias criteria for EPOC reviews*http://epoc.cochrane.org/sites/epoc.cochrane.org/files/uploads/14%20Suggested%20risk%20of%20bias%20criteria%20for%20EPOC%20reviews%202015%2009%2002.pdf). However, we acknowledge that these tools are designed simply to explore potential bias and, unlike Cochrane risk of bias tools for randomised and non-randomised studies, have not had their validity demonstrated. The remaining three categories of evidence are anticipated to make either a theoretical or contextual contribution, and therefore it is not meaningful to undertake a formal quality assessment of such items. For these conceptual and contextual types of evidence, we will go beyond the conventional focus on the methodological quality of studies to employ a more reflexive approach to locate the contribution of each item within the overall body of evidence and to assess its specific contribution, according to accepted principles of realist synthesis. Where evidence is considered to be of marginal rigour, we will iteratively and purposively seek additional confirmatory or disconfirming evidence. Thus we will combine the rigour of the systematic review process for category 1 materials with the acknowledged trade-off between relevance and rigour for the remaining categories of material [21], whilst being sure to clearly delineate between materials selected for rigour, for context and for conceptual contribution.

### Strategy for data synthesis

This synthesis will employ best fit framework synthesis, as a rapid tool by which to facilitate the data extraction and analysis process, combined with realist synthesis principles to maximise the value of the interpretative process resulting in practicable and feasible recommendations for practice. Dixon-Woods [30] suggests that framework synthesis is “especially suitable in addressing urgent policy questions where the need for a more fully developed synthesis is balanced by the need for a quick answer”. The best fit framework synthesis methodology was developed by Carroll et al. [31] as a pragmatic variation on framework synthesis. Best fit framework synthesis introduces the deductive step of developing an a priori framework, thus “harnessing the recognised strengths of both framework and thematic synthesis” [32]. Best fit framework synthesis will be combined with a synthesis following realist principles which are particularly suited to exploring what works, when, for whom and in what circumstances. The realist principles are derived from work by Pawson and Tilley [33], who recognised the need for methods suited to the inherent complexity within change programmes and their evaluation. The realist synthesis approach [34] has been consolidated and extended in more recent studies such as Rycroft-Malone et al. [35]. The realist approach acknowledges that interventions do not necessarily transfer easily from one setting to another and offers deeper insights into the contextual factors involved in change. Following synthesis, we will offer practitioner relevant dissemination activities to mobilise knowledge and support decision makers [36].

## Discussion

Vanguards face the challenge of identifying and designing interventions to deliver better value, improved outcomes and reduced utilisation in a climate of increasing financial pressures. MCPs, integrated care programmes and Vanguards are all examples of system change, and before embarking on such transformations, it is prudent to learn from existing research and practice, to facilitate understanding and improvements in local contexts.

Our synthesis is based around the delivery of useable summaries and tools which can support evidence-informed design, delivery and evaluation of new care models in health and social care. We will deliver a full evidence synthesis, including findings, conclusions, recommendations for further research (identification of knowledge gaps and uncertainties) and recommendations for practice. As described previously, this synthesis will employ best fit framework synthesis, as a rapid tool by which to facilitate the data extraction and analysis process, combined with realist synthesis principles to maximise the value of the interpretative process resulting in practicable and feasible recommendations for practice. This approach acknowledges and articulates contextual factors, thus providing the “thick descriptions” described by Polit and Beck [37] which help the target audience to understand what may be transferable to their specific contexts.

We will produce a range of outputs within the dissemination strategy, influenced by Colquhoun et al.’s [38] four key components for knowledge mobilisation as part of the dissemination:

- Strategies and techniques (active ingredients)—motivation, capability, opportunities
- How they function (causal mechanisms)—influenced by a variety of contextual factors
- How they are delivered or applied (mode of delivery)—e.g. face to face, brochure, mass media
- What they aim to change (intended targets)

As such our dissemination activities will comprise:

- A visual conceptual model, highlighting the key findings of the evidence synthesis to appeal to busy decision makers, which will align with the evaluation approach of the Vanguards
- A plain English summary which system leaders can use with local communities/patient and carer representatives
- A live web resource to share aims, objectives and methodology and in due course highlight key findings and recommendations
- Briefings for local health economies/decision makers, which incorporate key reflections to consider:
  - (Co-)designing new models of care (interventions)
  - Implementing new models of care (mechanisms)
  - Evaluating and measuring new models of care (outcomes)
  - Barriers and enablers (contextual factors)
- Social media promotion as relevant

Throughout the synthesis, the project team will be liaising with related projects under the same theme, to explore areas of synergy and to avoid unnecessary duplication. One such project, also focused on the MCP model, is seeking to identify learning from the international literature with the aim of “producing more strongly evidence-based logic models to inform development of MCPs”. (Rod Sheaff, Mark Pearson, Richard Byng, Helen Lloyd, Simon Briscoe, Jose Valderas-Martinez. From programme theory to logic models for multispecialty community providers: a realist evidence synthesis. PROSPERO 2016:CRD42016038900).

## Additional files

Additional file 1: Objectives of the Project Advisory Group. (PDF 181 kb) Additional file 2: Core set of potential search terms. (PDF 184 kb) Additional file 3: PRISMA-P checklist. (PDF 233 kb)

## Abbreviations

CIMO: Context, intervention(s), mechanism(s), outcome(s)
MCP: Multispecialty community provider
