# Supplementary material for: An evidence synthesis of the international knowledge base for new care models to inform and mobilise knowledge for multispecialty community providers (MCPs)
Source: Syst Rev. 2016 Oct 1;5:167. doi: 10.1186/s13643-016-0346-x (PMC5045634; doi:10.1186/s13643-016-0346-x)
Supplement: Additional file 1: — Objectives of the Project Advisory Group. (PDF 181 kb) [file 13643_2016_346_MOESM1_ESM.pdf]

**Additional file 1: Objectives of the Project Advisory Group**

The objectives of the Advisory Group are to:

- provide stakeholder perspectives on the scope of the synthesis and on emerging findings;
- inform the study design, including input into the search strategy;
- participate in strategic decisions about the project;
- advise on key policy, strategy and communication activities which may inform or impact this project;
- advise on the identification of key audiences and networks for dissemination activity;
- advise on dissemination formats, routes and activities;
- advise on interpretation of findings, to ensure relevance to stakeholders;
- support the translation of findings into actionable recommendations and tools for local health economies.
