# Supplementary material for: An evidence synthesis of the international knowledge base for new care models to inform and mobilise knowledge for multispecialty community providers (MCPs)
Source: Syst Rev. 2016 Oct 1;5:167. doi: 10.1186/s13643-016-0346-x (PMC5045634; doi:10.1186/s13643-016-0346-x)
Supplement: Additional file 2: — Core set of potential search terms. (PDF 184 kb) [file 13643_2016_346_MOESM2_ESM.pdf]

Additional File 2 : Core set of potential search terms

Features of MCP model	Potential search terms
Patient-centred coordinated care	PATIENT CENTERED CARE/, "collaborative care", "holistic care", "integrated care planning", "patient-centred care", coordinated care
GP-led out of hospital care	COMMUNITY HEALTH SERVICES/, GENERAL PRACTICE/, AMBULATORY CARE FACILITIES/
Enhanced primary care	"extend* primary care", "enhance* primary care"
Delivery to localities/communities/ neighbourhoods	hubs, clusters, "single point of access", localit*, communit*, neighbourhood*, "third sector integration"
Integrated community teams	DELIVERY OF HEALTH CARE, INTEGRATED/, "multidisciplinary team*", "team* without walls", "integrated community team*", PATIENT CARE TEAM/
Community mobilisation	mutuality, coproduction, "community resilience", "community asset-based approach", "social movement*", "community network*", "place-based commissioning", COMMUNITY NETWORKS
**Common activities**	**Potential search terms**
Extended GP access	"extended adj3 hours"
Care navigators/wellbeing support workers	"community anchor*", "volunteer health champion*", "peer support", "care navigator*", "wellbeing support worker*"
Community based specialists	"community consultant*", "community adj3 specialist*"
Patient activation/empowerment	SELF CARE/, "self management", PATIENT PARTICIPATION/, "patient adj3 empower*"
Prevention/proactive care	SECONDARY PREVENTION/, TERTIARY PREVENTION/, "disruptive prevention", "proactive care"
Outcomes based contracting	"outcome*-based contract*", "outcome* based commissioning", "incentive*", "currenc*"
Stratification	"risk stratif*", "case finding"
Population health	COMMUNITY HEALTH PLANNING/, "population health"
**Organisational factors**	**Potential search terms**
New and expanded workforce roles	HEALTH MANPOWER/, "expanded role*", "community pharmacist*", "physician assistant*", hospitalist*, "generic role*"
Governance and operating models	"accountable care organi*ation*", "accountable care system*", "primary care at scale", "federation*", "network*", "super-partnership*", "community health organi*ation*", "multi practice organi*ation*"
